# What We Expect Is Not Always What We Get: Evidence for Both the Direction-of-Change and the Specific-Stimulus Hypotheses of Auditory Attentional Capture

**DOI:** 10.1371/journal.pone.0111997

**Published:** 2014-11-13

**Authors:** Anatole Nöstl, John E. Marsh, Patrik Sörqvist

**Affiliations:** 1 Department of Building, Energy and Environmental Engineering, University of Gävle, Gävle, Sweden; 2 School of Psychology, University of Central Lancashire, Preston, United Kingdom; 3 Linnaeus Centre HEAD, Swedish Institute for Disability Research, Linköping University, Linköping, Sweden; Goldsmiths, University of London, United Kingdom

## Abstract

Participants were requested to respond to a sequence of visual targets while listening to a well-known lullaby. One of the notes in the lullaby was occasionally exchanged with a pattern deviant. Experiment 1 found that deviants capture attention as a function of the pitch difference between the deviant and the replaced/expected tone. However, when the pitch difference between the expected tone and the deviant tone is held constant, a violation to the direction-of-pitch change across tones can also capture attention (Experiment 2). Moreover, in more complex auditory environments, wherein it is difficult to build a coherent neural model of the sound environment from which expectations are formed, deviations can capture attention but it appears to matter less whether this is a violation from a specific stimulus or a violation of the current direction-of-change (Experiment 3). The results support the expectation violation account of auditory distraction and suggest that there are at least two different expectations that can be violated: One appears to be bound to a specific stimulus and the other would seem to be bound to a more global cross-stimulus rule such as the direction-of-change based on a sequence of preceding sound events. Factors like base-rate probability of tones within the sound environment might become the driving mechanism of attentional capture—rather than violated expectations—in complex sound environments.

## Introduction

Past experience is used to predict and prepare action in response to future events [1]. For instance, the auditory system extracts systematic regularities in the sound environment and uses this information to predict future sounds [2]–[3]. When expectations are violated, such as when a sound event deviates from the systematic regularities within the sound stream, the deviant event captures attention (i.e., the locus-of-attention is directed away from the ongoing task towards the sound), and interrupts the execution of the concurrent task [4]–[5]. The aim of the present study is to examine the nature of the cognitive representation that forms the basis of such expectations.

A common paradigm used in the field of behavioral auditory distraction research is the cross-modal auditory distraction paradigm [6]. In this paradigm, participants respond to visual targets that are preceded by an irrelevant tone. The same tone is repeated in most cases (standard trials), but occasionally the sequence of standard tones is interrupted by a different, deviating tone (a deviant trial). The response time to the visual target on deviant trials is typically longer than on standard trials, and the magnitude of this deviation effect is quantified by measuring the difference in response time for standard trials and for deviant trials.

Several competing accounts of the deviation effect have been offered. According to the base-rate probability account, the deviant captures attention because it is rare in the present sound context [7]–[8]. Another possibility is offered by the perceived local change account, whereby the perceived discrepancy between the deviant sound and the preceding standard sound captures attention [9]–[11]. Yet a third account suggests that the deviant captures attention because the participant is expecting a standard tone and the deviant violates this expectation [5]. Recent research suggests that violated expectations, rather than low base-rate probability and perceived local change, is the driving mechanism underpinning the deviation effect [4]–[5], [12].

To investigate how violated expectations contribute to the magnitude of the deviation effect, Nöstl et al. [4] used a modified version of the cross-modal auditory distraction paradigm. Instead of a single standard, three different standard tones (880 Hz, 660 Hz, and 440 Hz) were used and they were arranged in a regular, repetitive cross-trial sequence (i.e., 660-440-660-880-660-440-660-880-660…). Occasionally, either the 440 Hz standard tone or the 880 Hz standard tone was exchanged with a pattern deviant (either a 220 Hz tone or an 1100 Hz tone). The exchange resulted in either a small difference (i.e., a difference of 220 Hz) or a large difference (i.e., a difference of 660 Hz) between the pattern deviant and the standard tone it replaced. The key finding from the study was a cross-over interaction, demonstrating that a low-pitch deviant (220 Hz) captured attention to a greater degree when it replaced a high-pitch standard (880 Hz) in comparison with when it replaced a low-pitch standard (440 Hz), and a high-pitch deviant (1100 Hz) was more captivating when it replaced a low-pitch standard (440 Hz) in comparison with when it replaced a high-pitch standard (880 Hz). The experiment thus suggests that a greater difference between an expected tone and the actually presented tone results in a greater magnitude of attentional capture. Notably, the difference between the preceding standard tone and the pattern deviant was kept constant (always 440 Hz) as was the base-rate probability of the deviants, which suggests that violated expectations, rather than perceived local change or base-rate probability, underpins the magnitude of distraction.

Even though our previous investigation supports the violated-expectations account, it remains unclear what the participants expect of the upcoming sound stimuli based on what they have learned from previous experience with the sound environment. One possibility is that the participants expect a specific stimulus, and when this expected stimulus is exchanged with another stimulus, the discrepancy between the two exact tones is what determines the magnitude of attentional capture. We refer to this as the *specific-stimulus hypothesis*. Another possibility is that the participants expect a change—an increase or a decrease—in pitch from one sound element to the next, and a sound that violates this expected direction-of-change captures attention. We refer here to this regularity-violation account as the *direction-of-change hypothesis*. For example, when the high-pitch deviant (1100 Hz) replaces a low-pitch standard (440 Hz) in the standard sequence (660-440-660-880-660-440-…), the pattern deviant violates the expected direction-of-change (i.e., a decrease in pitch from the preceding 660 Hz standard to the upcoming 440 Hz standard), whereas when the low-pitch deviant (220 Hz) replaces the same low-pitch standard (440 Hz), the deviant does not violate the expected direction-of-change (i.e., a decrease in pitch from the 660 Hz standard). Hence, on the basis of these conditions, whether the deviant captures attention because it violates the expectation of a specific stimulus item or because the deviant violates the expectation of pitch change in a specific direction is impossible to determine, because the two are perfectly confounded.

**Figure 1:**
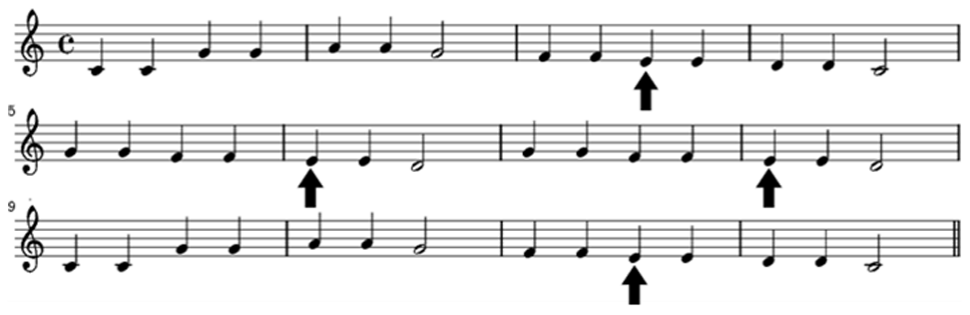
An illustration of ‘Twinkle, twinkle, little star’. Black arrows indicate sound sequence positions where tone replacements were occationally inserted during Experiment 1.

The purpose of the experiments reported here was to investigate whether unexpected auditory events capture attention because they violate the expectation of a specific stimulus, the expectation of a specific cross-stimulus pitch transition (direction-of-change), or both. To achieve this goal, we again utilized a modified version of the cross-modal auditory distraction paradigm. As the sound sequence has to be well-learned for the pattern deviant to capture attention [4]—and indeed be experienced as deviating—we decided to use a cross-trial tone sequence that comprised the well-known tune ‘Twinkle twinkle little star’ (Figure 1). The device of presenting a deviant note in a well-known melody is well established. Particularly it has proved useful in investigating double dissociations between brain responses to memory- and rule-based violations in melodies with the purpose of attempting to understand the neural basis of rule-governed and memory-based knowledge of music [13]. In the context of the current investigation, the use of a well-known melody, for which there is long-term knowledge, is both original and informative. For example, a well-known melody circumnavigates the problems associated with sequence learning of unfamiliar tonal patterns—whereby the auditory sequence must first be learnt before deviant notes can be recognized as such—and therefore the effects of note violations (or novels) can be observed early within the experimental session.

## Experiment 1

Experiment 1 was specifically designed to test the idea that a pattern deviant captures attention to a larger degree if the discrepancy in pitch between the expected tone and the deviant is large compared to when it is intermediate, even if both tones confirm the expected direction of pitch change. Moreover, Experiment 1 aimed to test if a pattern deviant that confirms the expected direction of pitch change still captures attention more than a pattern deviant that violates the expected direction of pitch change, as long as the former differs more than the latter from the expected stimulus item.

**Figure 2:**
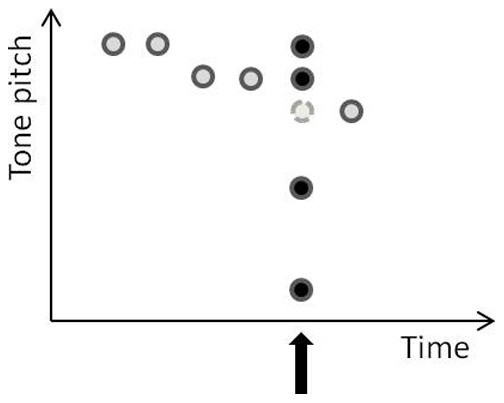
An illustration of the design of Experiment 1 and the possible tone replacement at the sequence position wherein the tone E5 would be presented in the original ‘Twinkle, twinkle, little star’ sound sequence. The light grey circle indicates the replaced tone (E5) and the black circles depict replacing/deviant tones. The set of replacing/deviant tones included F#5 (i.e., a small deviation from the replaced tone and an unexpected sequence-change direction), F5 (i.e., a perceptual change from the previous sound stimulus is held constant, but a small deviation from the replaced tone and an unexpected change direction), E4 (i.e., an intermediate deviation from the replaced tone, but a change in the expected direction) and E3 (i.e., a large deviation from the replaced tone, but a change in the expected direction).

The musical notes used in Experiment 1 were ‘A, C, D, E, F, G’ (all played in octave band 5). As shown in Figure 2, the ‘E5’ note, which always follows immediately after an ‘F5’, was occasionally replaced by one of four pattern-deviants (‘E3’, ‘E4’, ‘F5’, ‘F#5’). According to the specific-stimulus hypothesis, the pitch difference between the exact expected tone and the pattern-deviant should be the basis of the magnitude of attentional capture. Hence, this account predicts that ‘E3’ will be the most captivating pattern-deviant as it differs the most from the expectation of ‘E5’. On the other hand, according to the direction-of-change hypothesis, ‘F#5’ should be the most captivating pattern-deviant, as the participants should expect a drop in pitch after ‘F5’ and the presentation of ‘F#5’ instead of ‘E5’ violates this expectation. Pattern-deviants ‘E3’ and ‘E4’, in contrast, should not capture attention as they both confirm the expectation of a drop in pitch. In addition, we included a control condition wherein the perceived local change from the preceding stimulus to the pattern-deviant was held constant (i.e., when the pattern-deviant is ‘F5’). Moreover, all pattern-deviants had equal base-rate probability to rule out the possibility that differences in rarity contribute to the differences between conditions.

In our previous study [4], the sound-sequence was arbitrary and hence unfamiliar to the participants at the beginning of the experiment. In this context, there was a period of build-up before the pattern deviants began to capture attention (as they arguably were not experienced as pattern deviants at the beginning of the study). In the present study, the sound-sequence is familiar to the participants from the beginning of the study and therefore, in contrast to the previous study, we expected to see attention capture from pattern deviants already at the outset.

### Method

#### Participants

A total of 24 students at the University of Central Lancashire took part in this experiment. All reported normal or corrected-to-normal vision and normal hearing. They received a small honorarium for their participation. The study was approved by the University of Central Lancashire's Ethical Review Board. Participants gave both oral and written consent as is deemed sufficient by the Ethical Review Board when data are treated anonymously and whereby no apparent ethical research complications with participation are identified.

#### Materials

The oddball task was the same as that used in [14]. The participants were requested to respond to arrows randomly pointing either to the left (<<<) or to the right (>>>) by pressing the corresponding arrow key on a computer keyboard. The computer recorded the response time (RT) between the onset of the arrow and when the participant pressed a button. The arrow was visible for 600 ms and was then replaced by a 250 ms visual mask (###). Any key press that took place after the arrow had disappeared was considered a response error. Each arrow was immediately preceded by a 200 ms (10 ms rise and fall time) sinewave tone (the arrow was presented at the offset of the tone). The tones were normalized and presented binaurally through headphones (Sennheiser HD 202) at approximately 65 dB (A). The temporal distance between tones was 1050 ms (onset-to-onset). The tones comprised the musical notes ‘A, C, D, E, F, G’. They were arranged in a well-known pattern, ‘Twinkle, twinkle little star’. All notes in the standard sequence were presented in the octave band 5 (see Figure 1). Occasionally, the sequence was interrupted by replacing the tone ‘E5’ with one of four different pattern-deviants (see Figure 2): ‘E3’, ‘E4’, ‘F5’ or ‘F#5’. When a tone replacement took place, it was always after two consecutive presentations of ‘F5’. This specific tone constellation occurs four times during the ‘Twinkle, twinkle little star’ tone pattern (Figure 1). Which of these four positions that was subject to a tone substitution was pseudo-randomized across trial blocks.

#### Design and Procedure

A within-participant design was used. Participants sat alone in a silent room in front of a computer to which headphones were attached. The computer controlled stimulus presentation and recording of responses. In advance of testing, the participants were told to ignore all sounds, to use their dominant hand when responding to the arrows, and to respond as accurately and quickly as possible. To familiarize the participants with the task, they began with a training phase that contained a full sequence of the tune without pattern-deviants. The experimental session which followed directly after the practice phase, consisted of 40 blocks and every block contained a full playback of ‘Twinkle, twinkle little star’. Each block contained one pattern-deviant, resulting in a total of 10 occurrences per pattern-deviant across the experiment. The order of the four types of pattern-deviants was randomized, and each type was followed equally as often by left as by right pointing target arrows. There was a 5 sec pause between each block. In order to control for knowledge of the melody, the participants were asked to name the tune at the end of the experiment.

### Results and Discussion

All participants (100%) were able to correctly identify the melody as “Twinkle, twinkle little star”. There were 50 error/absent responses (out of 960 responses in total across all participants) on deviant trials. Hence, error rate was low (5.2%). A repeated-measures analysis of variance (ANOVA) revealed a significant main effect for type of deviant, *F*(3, 69)  = 3.31, *MSE* = .006, *p* = .031, η_p_^2^ = .12. Follow up tests displayed an increased error rate for the ‘E3’ pattern deviant (*M* = 6.25%, *SD* = 8.2%) compared to the ‘F#5’ pattern deviant (*M* = 2.5%, *SD* = 4.4%), *t*(23)  = −2.10, *p* = .045. There was also an increased error rate for the ‘E4’ pattern deviant (*M* = 8.6%, *SD* = 11.9%) compared to the ‘F5’ pattern deviant (*M* = 3.3%, *SD* = 5.6%) and compared to the ‘F#5’ pattern deviant (*M* = 2.5%, *SD* = 4.4%), *t*(23)  = 2.1, *p* = .045, and, *t*(23)  = 2.4, *p* = .025, respectively. Trials with incorrect responses were excluded from the response time analysis.

**Figure 3:**
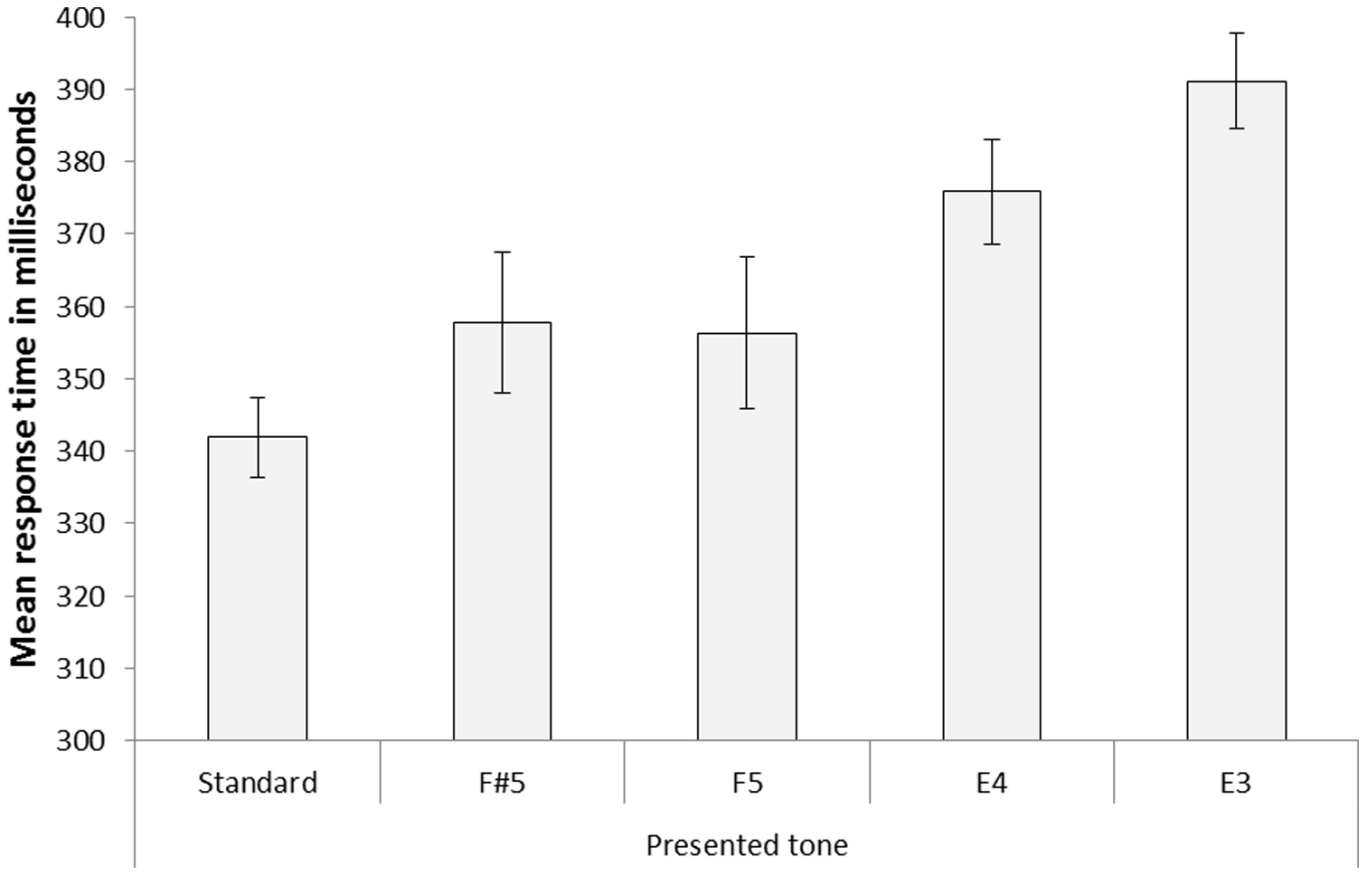
Mean response time data for standard trials and the four types of pattern deviants in Experiment 1. The deviants were F#5 (i.e., a small deviation from the replaced tone and an unexpected sequence-change direction), F5 (i.e., a perceptual change from the previous sound stimulus is held constant, but a small deviation from the replaced tone and an unexpected change direction), E4 (i.e., an intermediate deviation from the replaced tone, but a change in the expected direction) and E3 (i.e., a large deviation from the replaced tone, but a change in the expected direction). Error bars represent standard error of means.

The response time data for the four types of pattern deviants and for the standard (i.e., average response time to the arrow following the ‘F5’ note just before a pattern deviant) collapsed across all trial blocks are reported in Figure 3. A repeated-measures ANOVA with response time data as dependent variable and trial type as independent variable revealed a significant effect, *F*(4, 92)  = 11.47, *MSE* = 763.42, *p*<.001, η_p_^2^ = .33. The ‘E3’ pattern deviant (i.e., largest pitch difference from the replaced note, but in line with the current direction of change) captured attention to a larger degree than the ‘E4’ pattern deviant, *t*(23)  = 2.44, *p* = .023, the ‘F5’ pattern deviant, *t*(23)  = 3.29, *p* = .003, and the standard trials, *t*(23)  = 5.10, *p*<.001. Most importantly, the ‘E3’ pattern deviant also captured attention to a larger degree than the ‘F#5’ pattern deviant (i.e., relatively small pitch difference from the replaced note but inconsistent with the current direction of pitch change), *t*(23)  = 3.68, *p* = .001. Furthermore, the ‘E4’ pattern deviant also captured attention to a larger degree than the ‘F#5’ pattern deviant, *t*(23)  = 2.40, *p* = .025, and the difference between the ‘E4’ and ‘F5’ pattern deviants was marginal, *t*(23)  = 1.96, *p* = .065.

The results in Experiment 1 yield further support for the specific-stimulus hypothesis. The tone with the largest discrepancy (‘E3’) between the expected and the actually presented tone captured attention more strongly than the tone with intermediate discrepancy (‘E4’). Most importantly, the pattern deviant ‘E3’ captured attention to a greater degree than ‘F#5’, although ‘E3’ confirmed the expected directional pitch change and ‘F#5’ disconfirmed it, thus contradicting the direction-of-change hypothesis. Crucially, therefore, despite there being a deviation from the expected (downward direction of) pitch change with ‘F#5’, the extent of stimulus-mismatch from the expected/replaced item was more critical to distraction: ‘E3’ which was more disruptive, had the largest stimulus-mismatch from the expected/replaced sound, but this mismatch was in the expected downward direction of pitch change.

## Experiment 2

**Figure 4:**
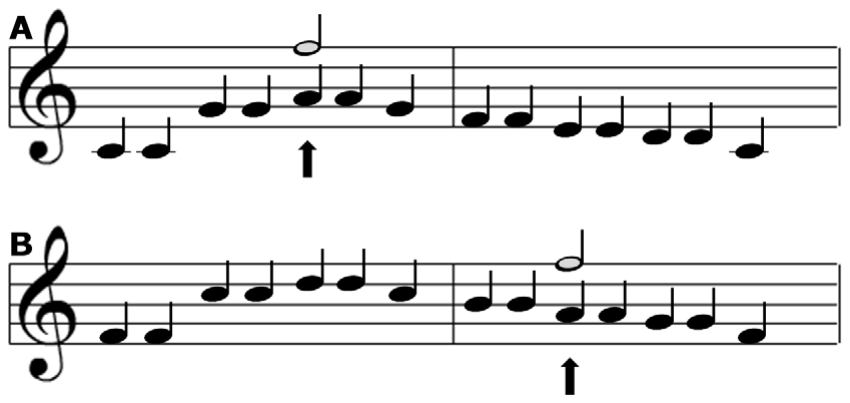
An illustration of the design in Experiment 2. The A5 note was ocassionally replaced by a deviant E6 tone (light grey note). Panel A depicts the condition in which the pitch of the deviant was in the expected direction of pitch transition, and Panel B displays the condition in which the pitch of the deviant was in the opposite (unexpected) direction.

Whereas Experiment 1 was primarily designed to test the specific-stimulus hypothesis, Experiment 2 was designed to optimize the conditions for finding support for the direction-of-change hypothesis. In order to investigate whether a violation to the direction-of-change captures attention over and above the difference between the expected and the actually presented tone, the expected tone has to (a) be preceded by either a higher tone (i.e., an expected fall in pitch) or a lower tone (i.e., an expected rise in pitch), and (b) the pitch difference between the expected tone and the preceding tone has to be held constant. This required tone constellation never occurs naturally in ‘Twinkle, twinkle little star’ and therefore a more general melody manipulation was deemed necessary. Specifically, this was accomplished by playing the first strophe of ‘Twinkle, twinkle little star’ in two different scales, either low or high (Figure 4).

To clarify, in Experiment 2, the tone ‘E6’ acted as the single deviant. The deviant occasionally replaced ‘A5’ regardless of which scale the melody was played. In the high-scale version of the tune, ‘A5’ was preceded by ‘Bb5’. In this case, the participants would be expecting a drop in pitch; a drop that would be confirmed by the standard tone ‘A5’ but violated by the deviant tone ‘E6’. In the low-scale version of the tune, on the other hand, ‘A5’ was preceded by ‘G5’. In this case, the participants would be expecting a rise in pitch; a rise that would be confirmed by the standard tone ‘A5’ and that also would be confirmed by the deviant tone ‘E6’. Support for the direction-of-change hypothesis would, hence, be obtained if there is a difference in the magnitude of attentional capture elicited by the pattern deviant depending on the scale context. Specifically, there should be an interaction between type of sound (standard vs. deviant) and the current direction of pitch change, because the deviant should be more captivating when it violates the current direction of pitch change, as opposed to when it does not violate the current direction of pitch change, according to the direction-of-change hypothesis.

### Method

#### Participants

A total of 35 students at the University of Gävle took part in this experiment. They all reported normal hearing and normal or corrected-to-normal vision and they received a small honorarium in exchange for participation. The study was approved by the Regional Ethical Review Board at the University of Uppsala (Dnr 2011/108). As the data would be treated anonymously, and no apparent ethical research complication with participation could be identified, oral consent was deemed sufficient by the Ethical Review Board. The data collector took note of the oral consent.

#### Materials

The oddball task was the same as in Experiment 1. The tones were arranged to resemble the first strophe of ‘Twinkle, twinkle little star’. In order to be able to investigate the direction-of-change the strophe could appear in either a high scale (F-Major) or a low scale (C-Major). The notes ‘A, C, D, E, F, G’ (all in octave band 5) constituted the strophe in the lower scale and the notes ‘A, Bb, C, D, G, F’ were used in the high scale version of the strophe. Occasionally, the sequence of standard tones was interrupted by replacing the first ‘A5’ note with a deviating tone, ’E6’ (see Figure 4).

#### Design and procedure

The design was similar to Experiment 1. The experimental session consisted of 20 blocks and each block consisted of one playback of the strophe without replacement followed by one playback with replacement. In every other block the playback scale was changed (i.e. from high to low and vice versa) and the order of the scales was counter balanced across participants. Each block contained one pattern-deviant, resulting in a total of 10 occurences per deviant (20 occurences of each if direction-of-change is ignored).

### Results and Discussion

As the strength of expectations rely on knowledge of the sound environment, only participants who managed to identify the tune as ‘Twinkle, twinkle little star’ should be included in the analysis. Fortunately, the melody was correctly identified as ‘Twinkle, twinkle little star’ by 100% of all participants.

Error rates were relatively low (7.1% for pattern deviants), and a paired samples *t*-test revealed no difference between the pattern deviant in the high tone scale context versus the pattern deviant in the low tone scale context, *t*(34)  = 0.39, *p* = .701. Only response times for correct responses were included in the response time analyses.

**Figure 5:**
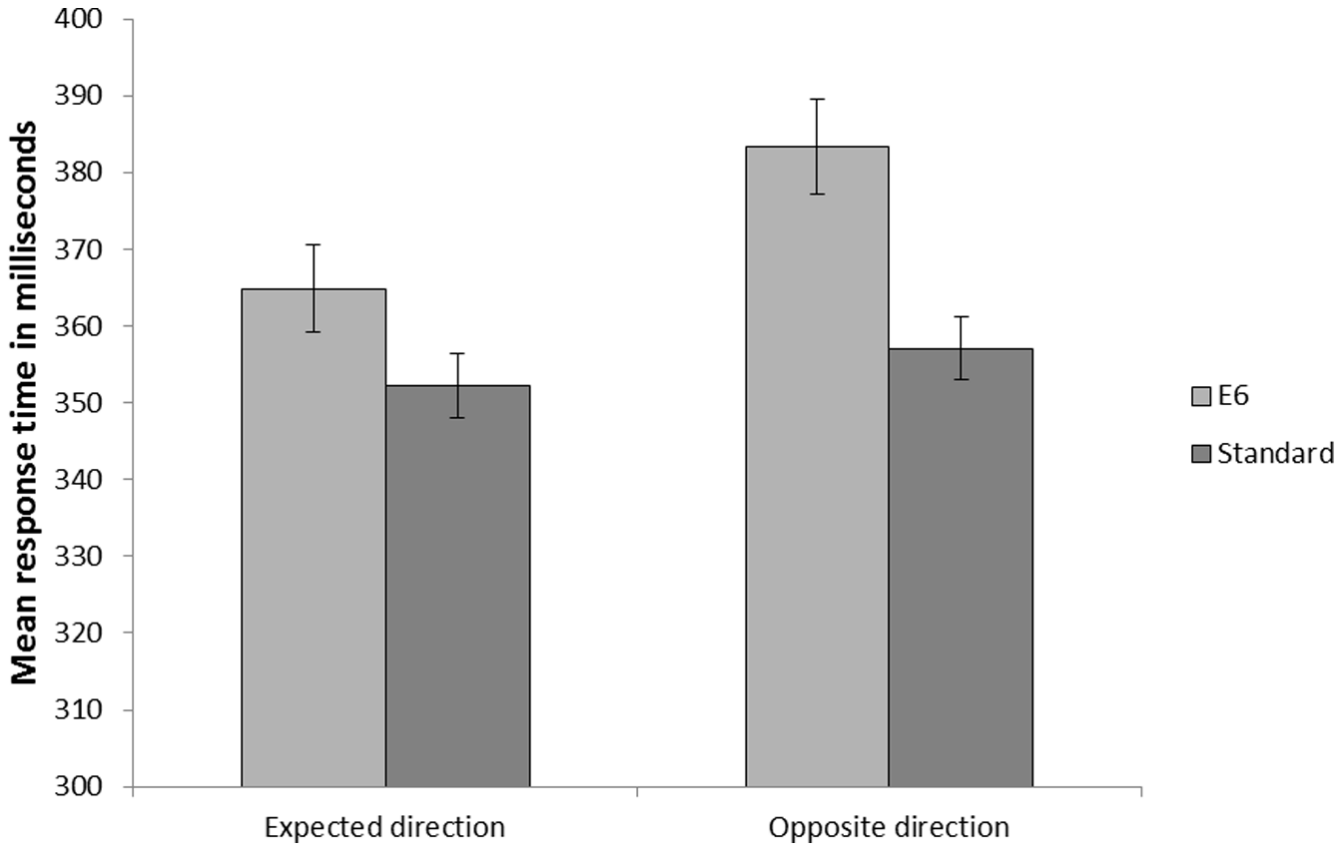
Mean response times in Experment 2. The pattern deviant E6 was more disruptive when presented in a tone scale context that made it violate the expected direction-of-pitch change. Error bars represent standard error of means.

As seen in Figure 5, the pattern deviant captured attention, and the magnitude of attentional capture was increased in the case where the pattern deviant violated the current directional pitch change compared to when it was consistent with it. These conclusions were supported by a 2(Sound: Standard vs. Deviant) ×2(Current direction of pitch change: Rise vs. Fall) repeated-measures ANOVA which revealed a main effect of Sound, *F*(1, 34)  = 31.52, *MSE* = 421.93, *p*<.001, η_p_^2^ = .48, and a main effect of Current direction of pitch change, *F*(1, 34)  = 17.86, *MSE* = 268.33, *p*<.001, η_p_^2^ = .34, and most importantly, a significant interaction between the factors was found, *F*(1, 34)  = 9.05, *MSE* = 179.33, *p* = .005, η_p_^2^ = .21. Follow up *t*-tests revealed an increased difference in response time between the pattern deviant and the standard tone when the direction of pitch was violated by the deviant (*M* = 26.31, *SD* = 26.73) compared to when the direction of pitch was not violated by the deviant (*M* = 12.68, *SD* = 22.11), *t*(34)  = 3.01, *p* = .005. Whereas the results in Experiment 1 lend support for the specific stimulus hypothesis, the results obtained in Experiment 2 reveal that the magnitude of attentional capture increases in the case of a violation of the direction of pitch change and therefore the results support the direction-of-change hypothesis.

## Experiment 3

The goal of Experiment 3 was to contrast the two types of expectations—the expectation of a specific stimulus and the expectation of a pitch transition—by including them simultaneously in a single sound environment. This was achieved by using a similar design as in Experiment 2. However, instead of using only one type of pattern deviant (‘E6’), a second pattern deviant (‘B6’) was also included. The chosen design allows for a thorough investigation concerning the relative strength of the different types of expectations. More specifically, it allows us to investigate if the different kinds of individual expectations prevail even though the number of parameters of the sound environment is increased or if their strength is weakened due to the complexity in the sound environment.

### Method

#### Participants

A total of 26 students at the University of Gävle took part in this experiment. They all reported normal hearing and normal or corrected-to-normal vision and they received a small honorarium in exchange for participation. The study was approved by the Regional Ethical Review Board at the University of Uppsala (Dnr 2011/108). As the data would be treated anonymously, and no apparent ethical research complication with participation could be identified, oral consent was deemed sufficient by the Ethical Review Board. The data collector took note of the oral consent.

#### Materials

**Figure 6:**
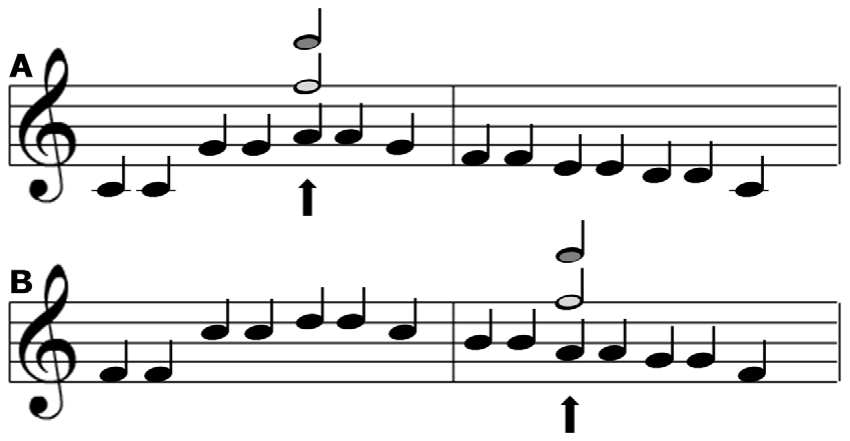
An illustration of the design in Experiment 3. The A5 note was either replaced by a E6 tone (light grey note) or a B6 tone (dark grey note). Panel A depicts the condition in which the pitch of the deviant was in the expected direction of pitch transition, and Panel B displays the condition in which the pitch of the deviant was in the opposite (unexpected) direction.

The oddball task was the same as in previous experiments and the tones were arranged in the same fashion as in Experiment 2. However, in Experiment 3, the sequence of standard tones was occasionally interrupted by replacing the first ‘A5’ note with one of two deviating tones, either ‘B6’ or ‘E6’ (see Figure 6).

#### Design and procedure

The design was similar to Experiment 2. The experimental session consisted of 40 blocks and each block consisted of one playback of the strophe without replacement followed by one playback with replacement. In every other block the playback scale was changed (i.e. from high to low and vice versa) and the order of the scales was counterbalanced across participants. Each block contained one pattern-deviant, resulting in a total of 10 occurences per deviant (20 occurences of each if direction-of-change is ignored).

### Results and Discussion

The melody was correctly identified as ‘Twinkle, twinkle little star’ by 100% of the participants. Error rates were low (3.8% for pattern deviants), and a 2(Sound:’E6’ vs. ‘B6’) ×2(Current direction of pitch change: Rise vs. Fall) repeated measures analysis of variance revealed a main effect of current direction of pitch change, *F*(1, 25)  = 5.44, *MSE* = 0.001, *p* = .028, η_p_^2^ = .18. Follow up *t*-tests confirm increased error rates when the pattern deviants were consistent with the current direction of pitch change, *t*(25)  = −2.33, *p* = .028. Only response times for correct responses were included in the response time analyses.

**Figure 7:**
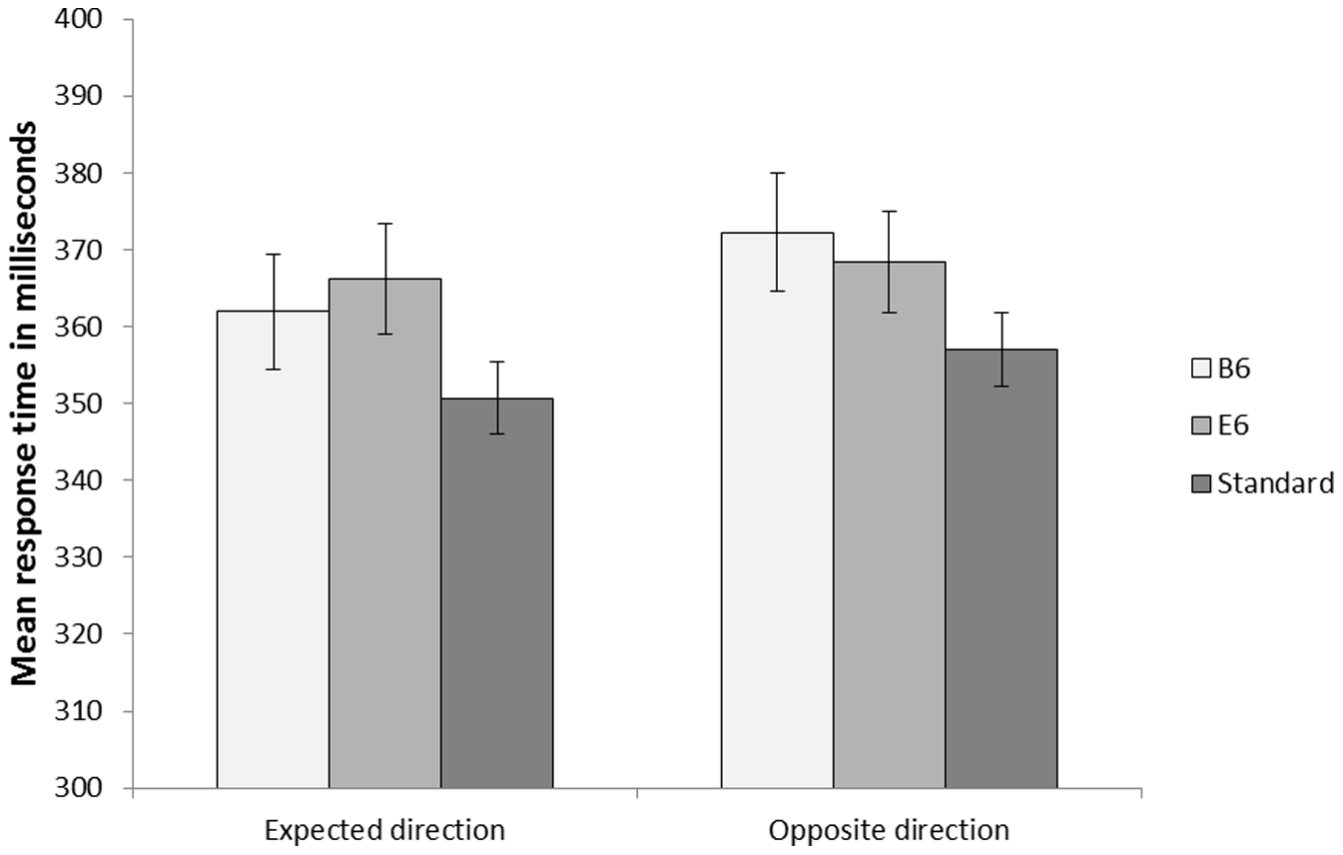
Mean response times in Experiment 3. ‘Expected direction’ is the condition where the strophe was played in C-major, whereas ‘Opposite direction’ is the condition where the strophe was played in F-major. Error bars represent standard error of means.

As can be seen in Figure 7, pattern deviants captured attention, and the difference between means were in line with the expected trend. However, the statistical analyses indicated that it did not matter whether the pattern deviant violated the current directional pitch change or whether the pattern deviant was consistent with the directional pitch change. Moreover, there is no observable difference between the two pattern deviants. These conclusions were supported by a 3(Sound: Standard vs. ’E6’ vs. ‘B6’) ×2(Current direction of pitch change: Rise vs. Fall) repeated measures ANOVA which revealed a main effect of Sound, *F*(2, 50)  = 10.21, *MSE* = 302.04, *p*<.001, η_p_^2^ = .29, and a main effect of Current direction of pitch change, *F*(1, 25)  = 6.03, *MSE* = 249.44, *p* = .021, η_p_^2^ = .19. However, no significant interaction between the factors was found, *F*(2, 50)  = 0.71, *MSE* = 305.10, *p* = .497, η_p_^2^ = .03. Follow up *t*-tests on the main effect of Sound revealed an increased mean response time for the ‘E6’ pattern deviant and the ‘B6’ pattern deviant compared to the standard tone, *t*(25)  = 3.93, *p*<.001, and *t*(25)  = 3.57, *p*<.001, respectively. However, no difference between the pattern deviants ‘E6’ and ‘B6’ was found, *t*(25)  = −0.08, *p* = .940.

As the frequentists approach cannot provide any likelihood for the null hypothesis, Bayesian inference was conducted to investigate the lack of interaction effect between Sound and Direction. The analysis revealed a Bayes Factor of 3.5 (positive evidence for H_0_). Hence, in the complex sound environment of Experiment 3, deviants capture attention, but it appears to matter less whether they violate an expectation of a specific stimulus or whehter they violate an expected pitch transition.

## General Discussion

The results showed that pattern-deviants capture attention to the degree they differ in pitch from the expected stimulus (Experiment 1), or when they violate the expected direction-of-pitch change (Experiment 2). In complex auditory environments, however, wherein it is difficult to rely on long-term memory of the sound environment and many different pattern-deviants are presented, deviants may capture attention, but whether they do so by diverging from a specific stimulus or by diverging from a direction-of-change appears less relevant (Experiment 3).

The present experiments were specifically designed to investigate the nature of the cognitive representations that are formed to make expectations of upcoming auditory stimulation. From the results obtained here, there appears to be at least two different expectations that can be violated. One appears to be bound to a specific stimulus (Experiment 1), the other would seem to be bound to a more global cross-stimulus rule such as the direction-of-change based on a sequence of preceding sound events (Experiment 2). This may be related to the distinction between stimulus-mismatch violation and regularity-violation: two mechanisms that appear to underpin attentional capture, at least in the context of the visual system [15]. Experiment 1 and 2 provided independent support for two concurrent mechanisms of attentional capture: one ‘local mechanism’ whereby sound captures attention when it violates a local rule (e.g., a rule about pitch regularities) and one more ‘global mechanism’ whereby sound captures attention when it violates a more global rule (that can be formed on the basis of the interrelationships between elements in the preceding sound stream, such as complex pitch transitions). Both mechanisms, however, may be influenced by the complexity of the auditory sequence and the capability to fashion a neural model representing these two aspects of the auditory environment. The efficient fashioning of a neural model that is sensitive to violation of specific stimulus expectations and the violation of pitch regularities may be particularly hindered in the context of complex auditory environments. Indeed, this is one possible explanation as to why the results of Experiment 3 supported neither the specific-stimulus (e.g., local mechanism or stimulus-mismatch) nor the direction-of-change (e.g., global mechanism or regularity) hypothesis. To elaborate further, the neural model required for deviance detection in Experiment 1 was relatively straightforward: The arrangements of notes within a well-known pattern (‘Twinkle twinkle little star’) in the same octave band. In this context the neural model receives support from top-down, schema-driven knowledge and it is readily apparent when a deviation occurs. In Experiment 2, the first strophe of ‘Twinkle twinkle little star’ was presented in high scale (F-Major) or low scale (C-Major). The scale within which the strophe was played was alternated from high to low, or vice versa. This increases the complexity of the neural model as not only might a representation of the sequence be fashioned due to top-down schema-driven processing of unattended sound [16], the same model (or a secondary neural model) may be created for the change from high to low, or low to high, changes between strophes. It is possible that direction-of-change as compared with specific-stimulus detection becomes the more important within this setting since changes in the scale of the Strophes represent changes in direction at a more global level: That is, the neural model could become sensitized to information within the environment that violate local direction-of-pitch transition as a consequence of the detection of global violations. Whereas the simple auditory environment in Experiment 1 and Experiment 2 facilitated the fashioning of a sensitive neural model, the results from Experiment 3 suggest that once a certain complexity threshold is crossed, by incorporating both local and global deviations, deviance detection switches from being a dynamic mechanism (i.e., sensitive to detecting differences among the deviations) to a static (or binary) mechanism (i.e., insensitive to specific details of the deviants).

Whilst it is well established that the neural model of an irrelevant sound sequence is based primarily upon the pre-categorical, acoustic properties of the irrelevant sequence, there is much less evidence that it represents post-categorical information. The results reported here are theoretically significant because they add to the small number of studies which demonstrate that long-term sequential rules form part of a neural model of irrelevant sound stimulation [13], [17]. Overall, the results support the expectation violation account of auditory distraction, whilst perceived local change and base-rate probability appear to be less important factors (Experiments 1 and 2). However, factors like base-rate probability might become more important in complex sound environments wherein it is difficult to fashion a neural model that is sensitive to specific details of deviants (Experiment 3).
